# *S*-allyl Cysteine Enhances Testosterone Production in Mice and Mouse Testis-Derived I-10 Cells

**DOI:** 10.3390/molecules26061697

**Published:** 2021-03-18

**Authors:** Md Masud Rana, Kota Shiozawa, Katsuyuki Mukai, Katsuhiko Takayanagi, Koichi Eguchi, Halima Sultana, Yusuke Ohsaki, Michio Komai, Hitoshi Shirakawa

**Affiliations:** 1 Laboratory of Nutrition, Graduate School of Agricultural Science, Tohoku University, 468-1 Aramaki Aza Aoba, Aoba-ku, Sendai 980-8572, Japan; masudrana@g-mail.tohoku-university.jp (M.M.R.); k.shiozawa@g-mail.tohoku-university.jp (K.S.); sultana.halima.d4@tohoku.ac.jp (H.S.); yusuke.ohsaki.a4@tohoku.ac.jp (Y.O.); mkomai@m.tohoku.ac.jp (M.K.); 2 R&D, Business Strategy, Healthcare SBU, Daicel Corporation, 2-18-1, Konan, Minato-ku, Tokyo 108-8230, Japan; kt_mukai@jp.daicel.com; 3 Arai Plant, Daicel Corporation, 1-1, Shinko-cho, Myoko 944-8550, Japan; kt_takayanagi@jp.daicel.com; 4 Innovation and Business Development Headquarters, Daicel Corporation, 1-8-23, Konan, Minato-ku, Tokyo 108-0075, Japan; ki_eguchi@jp.daicel.com; 5 International Education and Research Center for Food Agricultural Immunology, Graduate School of Agricultural Science, Tohoku University, 468-1 Aramaki Aza Aoba, Aoba-ku, Sendai 980-8572, Japan

**Keywords:** *S*-allyl cysteine, testosterone, protein kinase A

## Abstract

Hypogonadism, associated with low levels of testosterone synthesis, has been implicated in several diseases. Recently, the quest for natural alternatives to prevent and treat hypogonadism has gained increasing research interest. To this end, the present study explored the effect of *S*-allyl cysteine (SAC), a characteristic organosulfur compound in aged-garlic extract, on testosterone production. SAC was administered at 50 mg/kg body weight intraperitoneally into 7-week-old BALB/c male mice in a single-dose experiment. Plasma levels of testosterone and luteinizing hormone (LH) and testis levels of proteins involved in steroidogenesis were measured by enzymatic immunoassay and Western blot, respectively. In addition, mouse testis-derived I-10 cells were also used to investigate the effect of SAC on steroidogenesis. In the animal experiment, SAC significantly elevated testosterone levels in both the plasma and the testis without changing the LH level in plasma and increased phosphorylated protein kinase A (p-PKA) levels. Similar results were also observed in I-10 cells. The findings demonstrating the increasing effect of SAC on p-PKA and mRNA levels of *Cyp11a* suggest that SAC increases the testosterone level by activating the PKA pathway and could be a potential target for hypogonadism therapeutics.

## 1. Introduction

Testosterone is predominantly produced in Leydig cells of the testes [1]. Its synthesis depends on the release of luteinizing hormone (LH) from the pituitary gland, which requires gonadotropin-releasing hormone from the hypothalamus. In Leydig cells, LH binds to the luteinizing hormone receptor, a G-protein-coupled receptor that activates adenylate cyclase (AC) and enhances intracellular cAMP, which activates protein kinase A (PKA) and cAMP response element-binding protein (CREB). Subsequently, cholesterol is transported to the inner mitochondrial membrane by steroidogenic acute regulatory protein (StAR), converted to pregnenolone by CYP11A1, and eventually converted to testosterone by other steroidogenic enzymes [2].

Over-aging males develop late-onset hypogonadism, where Leydig cells gradually reduce their capacity to produce testosterone, and consequently, the blood testosterone level also declines [3]. A previous study showed that the testosterone levels in males start to decrease from their middle age by 2% per year [4]. Apart from the primary role of testosterone as a sex hormone in the male reproductive system, low testosterone levels are associated with the development of many diseases such as osteoporosis, type 2 diabetes, and cardiovascular diseases [5]. Low testosterone levels are also associated with obesity, depression, fatigue, reduced muscle mass, and loss of cognitive function [6]. Therefore, it is particularly important to maintain physiological testosterone levels to sustain a healthy life. To overcome hypogonadism or age-related testosterone decline in males, testosterone replacement therapy (TRT) has been a popular medication for several decades [7]. However, its applicability has raised some controversy, as a few cases of cardiovascular events have been reported, followed by TRT [8,9], warranting the search for alternative resources, such as nutritional supplements or natural compounds in diets that have the potential to boost testosterone levels. In this direction, several natural compounds that have the potential to enhance testosterone production either in vitro or in vivo have been explored [10,11,12,13,14]. For instance, vitamin K2 homolog menaquinone-4, geranylgeraniol, and cysteine sulfoxide have been shown to enhance testosterone levels via PKA activation [11,12,13]. Moreover, numerous flavonoids and isoflavonoid molecules have also been reported to have steroidogenic effects on Leydig cell lines or animal-based models [10,14]. In addition, ginger, onion, and honey have been demonstrated to have a testosterone-elevating effect in animal-based studies [15,16,17].

**Figure 1:**
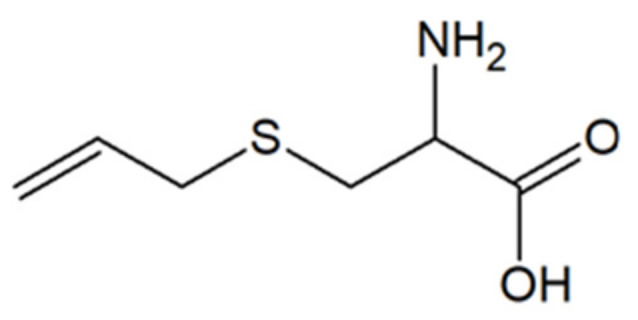
Structure of *S*-allyl cysteine.

*S*-allyl cysteine (SAC) (Figure 1) is a water-soluble organosulfur compound found in fresh garlic in little amount and is most abundant in aged garlic extract [18,19]. SAC is a potential antioxidant, anti-inflammatory, and anticancer agent [20,21]. Numerous studies have suggested that SAC ameliorates many diseases, such as cardiovascular disease, diabetes mellitus, and hypertension [22,23,24,25]. Furthermore, a pharmacokinetic study of SAC in animals such as rats and dogs revealed that SAC has high oral bioavailability, limited metabolism, and extensive renal reabsorption [26]. For the treatment of hypertension in human subjects, SAC in garlic extract revealed no apparent toxicity [27]. Moreover, garlic has been used as a traditional medicine and consumed as a spice since ancient times [19].

Recent studies suggest the protective role of SAC against oxidative damage of the male reproductive organ—SAC restored erectile dysfunction in a diabetes-induced rat model by reducing reactive oxygen species production [28]. It has also been shown that SAC could improve the number, motility, and DNA synthesis in sperm and reduce the oxidation marker protein in an old rat model [29]. Additionally, the potential of SAC to improve motility, plasma membrane integrity, and mitochondrial activity in spermatozoa was also documented in boar [30]. However, its effects on testosterone production have not been explored. Therefore, in this study, we investigated the effect of SAC on testosterone production in mouse testes and in mouse testis-derived I-10 tumor cells. The results suggested that SAC might activate PKA independent of cAMP for its steroidogenic effect.

## 2. Results

### 2.1. Effect of SAC on Testosterone Production in Testes and Plasma of Mice

**Figure 2:**
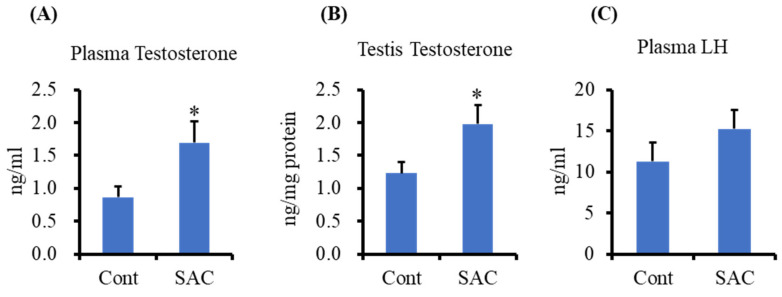
*S*-allyl cysteine (SAC) enhances testosterone production in mice. BALB/c mice were administered SAC at 50 mg/kg body weight intraperitoneally for 6 h. Testosterone level in (**A**) plasma (*n* = 6–7) and (**B**) testis (*n* = 7–8) was measured by enzyme immunoassay (EIA). (**C**) Plasma luteinizing hormone (LH) was measured in plasma by ELISA (*n* = 6). Data are presented as the mean ± standard error (SE). Data were analyzed by Student’s *t*-test. * *p* < 0.05 compared to the control group.

Testosterone levels in mouse testes and plasma were measured using the enzyme immunoassay (EIA) method. Plasma testosterone levels in the plasma of SAC-treated mice significantly increased compared to the controls (Figure 2A). Testosterone levels in the testes, the primary site for its synthesis, were significantly higher in SAC-treated mice (Figure 2B). However, there was no change in the plasma LH levels in the SAC-treated group compared to the control group (Figure 2C). These results suggest that SAC might work locally on Leydig cells of the testes rather than targeting the hypothalamus and pituitary gland.

### 2.2. Effect of SAC on Steroidogenic Protein Levels in the Testes of Mice

**Figure 3:**
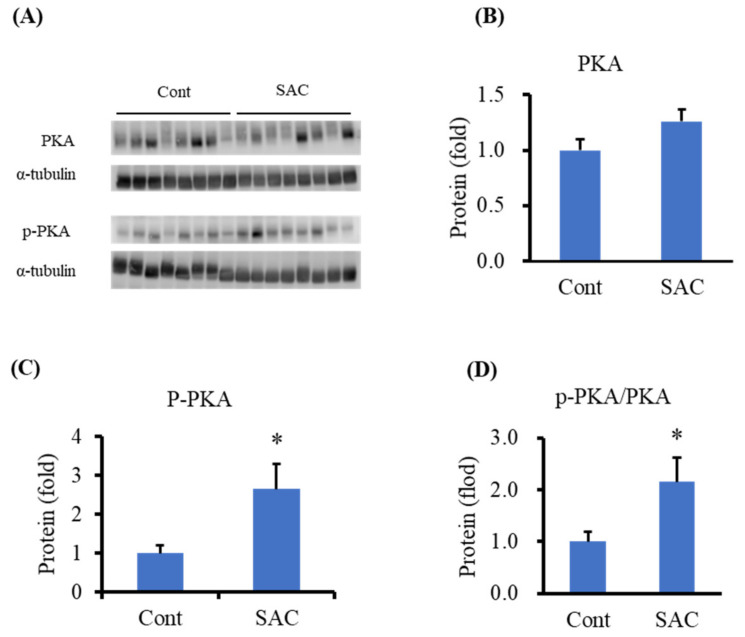
SAC activates protein kinase A (PKA) in the testes of mice. (**A**) Western blot image of protein expression of PKA and phosphorylated PKA (p-PKA) in the testis. (**B**–**D**) Quantification of protein expression of PKA, p-PKA, and p-PKA/PKA. Data are presented as the mean ± SE (*n* = 8). Data were analyzed by Student’s *t*-test. * *p* < 0.05 compared to the control group.

Next, we analyzed the levels of proteins involved in testosterone synthesis in the testis. We found that PKA levels did not change after SAC treatment, whereas the phosphorylated PKA (p-PKA) level was significantly higher in the SAC-treated group (Figure 3) than in the control group. The ratio of p-PKA to total PKA was also significantly high in the SAC-treated group. These results suggest that SAC might enhance testosterone production by activating PKA.

### 2.3. Effect of SAC on Testosterone Production in I-10 Cells

**Figure 4:**
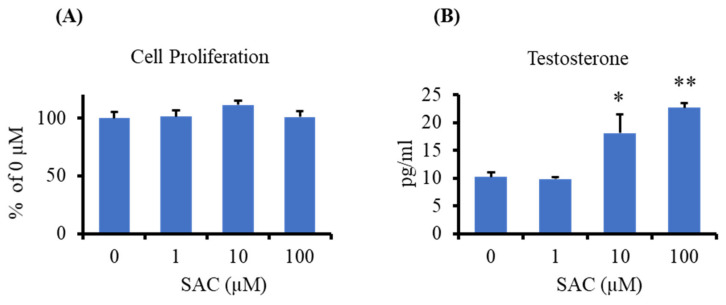
SAC enhances testosterone production in I-10 cells. (**A**) Cells at a density of 3.6 × 10^4^ cells/well were incubated in 96-well plates for 24 h with different concentrations of SAC (0, 1, 10, and 100 µM). Cell proliferation was determined by the water-soluble tetrazolium salts-1 (WST-1) assay. Data are presented as the mean ± SE (*n* = 4–5). Data were analyzed by one-way ANOVA. (**B**) Cells at a density of 6 × 10^4^ cells/well were incubated in 12-well plates with SAC for 24 h. Testosterone was measured from the medium by the EIA method. Data are presented as the mean ± SE (*n* = 3). Data were analyzed by Dunnett’s test. * *p* < 0.05 and ** *p* < 0.01 vs. 0 µM.

To investigate the direct effect of SAC on steroidogenesis, we used a mouse testis-derived I-10 cell line. First, we checked the cytotoxic or proliferative effect of SAC on I-10 cells using the water-soluble tetrazolium salts-1 (WST-1) assay. We observed that SAC has no cytotoxic or proliferation effect at a concentration of 1–100 µM on I-10 cells after 24 h of incubation (Figure 4A). Next, we measured the testosterone levels in I-10 cells treated with SAC at concentrations of 1, 10, and 100 µM for 24 h. Testosterone was measured from the culture medium by the EIA method. The results showed that SAC significantly enhanced the testosterone level at a concentration of 10 µM, whereas at a higher concentration (100 µM), it enhanced testosterone levels by approximately two times higher than that of the control group (Figure 4B).

### 2.4. Effect of SAC on the Activation of PKA in I-10 Cells

**Figure 5:**
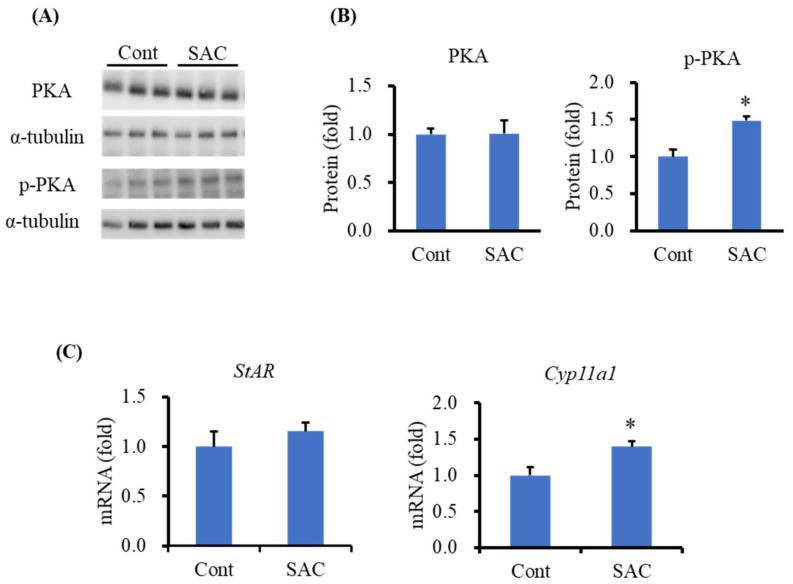
SAC activates PKA in I-10 cells. (**A**) Western blot image of PKA and p-PKA in I-10 cells. Cells (2 × 10^6^) were incubated for 1.5 h with 100 µM of SAC. Protein expression in the cell lysate was determined by the Western blot method. (**B**) Quantification of PKA and p-PKA protein expression in I-10 cells. (**C**) The mRNA expression level of *StAR* and *Cyp11a1* in I-10 cells. I-10 cells at a density of 10^6^ cells per 6 cm dish were treated with 100 µM of SAC for 3 h. mRNA expression was measured by qRT-PCR. Data are presented as the mean ± SE (*n* = 3). Data were analyzed by Student’s *t*-test. * *p* < 0.05 compared to the control group.

Next, we investigated the expression levels of PKA and p-PKA in SAC-treated I-10 cells to elucidate the effect of SAC on the activation of PKA in I-10 cells. We observed that SAC did not change the PKA level but significantly enhanced p-PKA expression in I-10 cells (Figure 5A,B), which was consistent with the results of the animal experiment (Figure 3; Section 2.2). Furthermore, we analyzed the mRNA expression levels of *StAR* and *Cyp11a1* in I-10 cells by quantitative RT-PCR. We observed that SAC significantly increased the mRNA level of *Cyp11a1* but did not change the *StAR* mRNA level after the indicated incubation time (Figure 5C).

## 3. Discussion

In this study, we used BALB/c mice as an animal model to study the effect of SAC on testosterone production. We found that SAC elevates testosterone levels in both the testes and plasma of BALB/c mice after a single intraperitoneal administration. As testosterone synthesis depends on the secretion of LH from the pituitary gland into the circulation [31], we measured LH levels in the plasma of mice. We found that SAC did not change the LH level in the plasma of mice. These results suggest that SAC might act directly on the testosterone-producing organ, the testis, rather than acting on the hypothalamus–pituitary axis. To confirm the direct role of SAC in testosterone synthesis, we used the testis-derived tumor cell line I-10, which secretes testosterone into the culture medium. We found that SAC enhances testosterone secretion from I-10 cells without any cytotoxic or cell proliferation effect at the indicated concentration. Taken together, these results from animal- and cell-based experiments confirm that SAC directly enhances testosterone production in Leydig cells.

Testosterone synthesis in Leydig cells is tightly regulated by a complex mechanism in which PKA plays a pivotal role. Activated PKA promotes phosphorylation of CREB and StAR proteins, which are indispensable for testosterone synthesis [32]. Therefore, in this study, we measured PKA and p-PKA levels in both animal- and cell-based experiments. We found that SAC enhances p-PKA levels in both mice and I-10 cells without enhancing PKA levels. Activation of PKA during testosterone synthesis can be performed in a cAMP-dependent and a cAMP-independent manner [33]. Hence, we measured intracellular cAMP levels in I-10 cells and found that SAC has no effect on cAMP production (data not shown). This suggests that activation of PKA by SAC might follow a cAMP-independent mechanism. Furthermore, we measured the mRNA levels of *StAR* and *Cyp11a1* in SAC-treated I-10 cells and found that SAC can enhance the mRNA levels of *Cyp11a1* but not *StAR* after the indicated incubation time in I-10 cells.

The CYP11A1 protein, also known as the P450 cholesterol side-chain cleavage enzyme, catalyzes the first rate-limiting step of steroid hormone synthesis, which involves converting cholesterol to pregnenolone in the mitochondria [34]. Pregnenolone is the precursor of all steroid hormone synthesis and is considered the primary neurosteroid in the brain [35]. In the brain, pregnenolone and other pregnenolone-derived neurosteroids have been reported to have neuroprotective effects, such as neuronal cell survival, memory function, and cognition [36]. In this study, we found that SAC enhances PKA activation and *Cyp11a1* gene expression in I-10 cells. Therefore, it can be inferred that SAC might have a steroidogenic effect on other organs such as the adrenal gland and brain by activating this pathway, although SAC has already been reported to have a neuroprotective effect on isolated hippocampal neuron cell culture, in addition to amelioration of memory function and depression in animal models by other mechanisms [37,38,39,40].

The StAR protein transfers cholesterol from the outer mitochondrial membrane to the inner mitochondrial membrane to the CYP11A1 protein for pregnenolone synthesis in steroidogenic cells [33]. The StAR protein can be phosphorylated by PKA for its maximal action, although StAR-independent cholesterol transfer to the mitochondria has been reported for steroidogenesis [41]. Either phosphorylation or transcription of *StAR* by different PKA isoforms has also been reported in steroidogenic cells [42]. However, the regulation of the StAR protein by activated PKA in I-10 cells has not been elucidated yet. In our cell-based study, we did not find significant change in *StAR* mRNA expression after SAC treatment at the indicated incubation time. Therefore, how the StAR protein is regulated in I-10 cells warrants further study.

The pharmacokinetics of SAC have been extensively investigated in animal models like rats and dogs. Two pharmacokinetic studies have demonstrated that SAC can metabolize to its N-acetylated form and to a lesser extent its sulfoxide form in rats [26,43]. Although the N-acetylated form of SAC and *S*-allyl cysteine sulfoxide (alliin) have not been reported to have a steroidogenic effect in vivo or in vitro, structurally similar compounds like propenyl-1-cysteine sulfoxide (isoalliin) and cycloalliin from onion extract have been reported to have a steroidogenic effect in I-10 cells [13]. Moreover, onion juice, which is rich in cysteine sulfoxides, showed a steroidogenic effect in animal-based experiments [44]. Therefore, it is probable that SAC, along with other cysteine derivatives, could be metabolized to other functional compounds for steroidogenesis, thereby behaving as a prodrug.

Collectively, the findings of this study show that SAC can be used as a dietary supplement and has therapeutic potential to prevent and treat age-related testosterone decline in males. However, the mechanism of testosterone production by SAC via activation of PKA and CYP11A1 should be elucidated by further in-depth investigation.

## 4. Materials and Methods

### 4.1. Materials

SAC was purchased from the Tokyo Chemical Industry Co., Ltd. (Tokyo, Japan). Mouse testis-derived I-10 tumor cells were obtained from the Health Science Research Resource Bank (Osaka, Japan).

### 4.2. Animals

Six-week-old male BALB/c mice were purchased from CLEA Japan, Inc. (Tokyo, Japan). The mice were maintained for 1 week in a 12 h/12 h dark/light cycle and had free access to the F-2 laboratory diet (Funabashi Farm Co., Funabashi, Japan) and tap water. SAC at a concentration of 50 mg/kg body weight in saline was administered to mice (7 weeks of age) intraperitoneally in the treatment group (*n* = 8), and the control group (*n* = 8) was injected with only saline. After 6 h, the mice were euthanized, blood was collected from the heart using a heparinized syringe, and the testes were dissected for further analysis.

### 4.3. Cell Culture

I-10 cells were maintained in Ham’s F-10 medium (Sigma-Aldrich, St. Louis, MO, USA) supplemented with 10% fetal bovine serum (Cosmo Bio Co., Ltd., Tokyo, Japan), 50 U/L of penicillin, and 50 mg/mL of streptomycin (Gibco, Thermo Fisher Scientific, Carlsbad, CA, USA) in a 10 cm dish in a humidified chamber at 37 °C and 5% CO_2_. The cells were used for the experiments when they reached 70–80% confluence. SAC was dissolved in water at a concentration of 200 mM and kept at −20 °C (stock solution). The stock solution was further diluted with water before mixing with the medium. The final concentration of water was 0.1% in the medium used for the experiments.

### 4.4. Testosterone and Luteinizing Hormone Measurement

Testosterone was measured using EIA according to the manufacturer’s instructions (Cayman Chemical Co., Ann Arbor, MI, USA). Briefly, testes were homogenized in phosphate buffered saline (PBS) (100 mg testes: 5 mL PBS). Next, 1 mL of the testis homogenate or 200 µL of plasma was mixed with 5 times’ volume of diethyl ether and centrifuged at 1500× *g* for 3 min. Approximately 90% of the upper ether layer was collected in another tube. This procedure was repeated three more times, and the collected ether layer was evaporated using a vacuum evaporator (Spin Dryer Light VC-36R, TAITEC Corp., Saitama, Japan). After evaporation, the EIA buffer supplied with the kit was added to the residue and testosterone was measured from this solution. The protein amount from the testis homogenate was measured by the Lowry method [45]. Testosterone levels in the testes were normalized to protein levels. For cell-based experiments, the medium was centrifuged at 1000× *g* for 5 min, and the supernatant was collected. Testosterone levels in the supernatant were measured using the EIA method. Luteinizing hormone levels in the plasma were measured by ELISA according to the manufacturer’s instructions (Endocrine Technologies, Newark, NJ, USA).

### 4.5. Cell Proliferation Assay

I-10 cells at a density of 3.6 × 10^4^ cells per well in 96-well plates were incubated overnight, followed by replacing the medium with fresh medium containing 1, 10, and 100 µM of SAC. After 24 h of incubation, WST-1 reagent (Takara Bio Inc., Shiga, Japan) was added to the medium. Absorbance at different time intervals was measured at 450 nm using a microplate reader XR (Bio-Rad, Hercules, CA, USA), and the proliferation rate was calculated from the absorbance data.

### 4.6. Western Blot

I-10 cells were collected by scraping in lysis buffer (50 mM Tris-HCl at pH 7.5, 150 mM NaCl, 0.1% SDS, 5 mM EDTA) containing phosphatase inhibitor and protease inhibitor (Roche Applied Science, Mannheim, Germany). For animal samples, the testes were homogenized with PBS containing phosphatase inhibitor and protease inhibitor. Proteins were denatured in gel-loading buffer, and approximately 15 µg of proteins were resolved in 10–20% SDS-polyacrylamide gel (Wako Pure Chemical Industries, Osaka, Japan) by electrophoresis. Proteins separated from the gel were transferred into polyvinylidene difluoride membranes (Millipore, Billerica, MA, USA). The membranes were blocked for 2 h in TBS-T buffer (10 mM Tris-HCl at pH 7.5, 150 mM NaCl, and 0.1% Tween 20) containing 5% skim milk or 3% bovine serum albumin. The membranes were then incubated overnight with blocking buffer containing an antibody against PKA, and phosphorylated PKA (Cell Signaling Technology, Danvers, MA, USA), followed by incubation for 1 h with horseradish peroxidase (HRP)-tagged secondary antibody. Antibodies against α-tubulin (Sigma-Aldrich) were incubated for 1 h followed by HRP-tagged secondary antibody. The immunoreactive band was detected using Immobilon Western Detection Reagent (Millipore) and visualized using a LAS-4000 mini luminescent image analyzer (Fujifilm, Tokyo, Japan). Relative protein expression levels were measured by normalizing with the expression of α-tubulin or β-actin.

### 4.7. RNA Extraction and mRNA Quantification

Total RNA was extracted from the cells using Isogen reagent (Nippon Gene, Tokyo, Japan) according to the manufacturer’s instructions. RNA purity and quantity were measured spectrophotometrically using a NanoDrop spectrometer (NanoDrop Technologies, Wilmington, DE, USA) from the absorbance at 260 nm in relation to that at 280 nm. RNA (4 µg) was used as a template for cDNA synthesis. RNA was denatured at 65 °C for 5 min with 2.5 µM oligo-dT primer (Hokkaido System Science Co., Sapporo, Japan) and 0.5 mM dNTP (GE Healthcare, Tokyo, Japan). cDNA was synthesized from the denatured RNA using 50 U SuperScript III reverse transcriptase (Invitrogen, Carlsbad, CA, USA) and 20 U RNaseOUT RNase inhibitor (Invitrogen) in RT buffer (50 mM Tris-HCl at pH 8.3, 75 mM KCl, 3 mM MgCl_2_, and 5 mM dithiothreitol) at 50 °C for 60 min. An aliquot of this cDNA was used as a template to amplify the target sequence using gene-specific primers (Table 1) and SYBR Premix Ex Taq solution (Takara Bio, Otsu, Japan). Quantitative RT-PCR was performed using the CFX96 Touch Real-Time PCR Detection System (Bio-Rad Laboratories Inc., Hercules, CA, USA). The mRNA levels were then normalized to the levels of eukaryotic elongation factor 1α1 (*Eef1a1*).

### 4.8. Statistical Analysis

Data are presented as the mean ± standard error (SE). Statistical analysis was performed using Student’s *t*-test or one-way ANOVA, followed by Dunnett’s test using SigmaPlot version 12.5 (Systat Software Inc., San Jose, CA, USA). All statistical analyses were conducted with a significance level of α = 0.05 (*p* < 0.05).

## Figures and Tables

**Table 1:** Nucleotide sequence of the primers used for quantitative RT-PCR.

Gene Name	Forward Primer	Reverse Primer
*Cyp11a1*	5′-CGTGACCTTGCAGAGGTACACT-3′	5′-GCTGGAATCTTGTAATTACGAAGCA-3′
*StAR*	5′-GGAGCTCTCTGCTTGGTTCTC-3′	5′-TTAGCACTTCGTCCCCGTTC-3′
*Eef1a1*	5′-GATGGCCCCAAATTCTTGAAG	5′-GGACCATGTCAACAATTGCAG-3′

## Data Availability

Data are contained within the article.

## Sample Availability

Samples of the compounds are not available from the authors.

## References

[1] Zirkin B.R., Papadopoulos V. (2018). Leydig cells: Formation, function, and regulation. Biol. Reprod..

[2] Miller W.L., Auchus R.J. (2011). The Molecular Biology, Biochemistry, and Physiology of Human Steroidogenesis and Its Disorders. Endocr. Rev..

[3] Nieschlag E. (2020). Late-onset hypogonadism: A concept comes of age. Andrology.

[4] Feldman H.A., Longcope C., Derby C.A., Johannes C.B., Araujo A.B., Coviello A.D., Bremner W.J., McKinlay J.B. (2002). Age Trends in the Level of Serum Testosterone and Other Hormones in Middle-Aged Men: Longitudinal Results from the Massachusetts Male Aging Study. J. Clin. Endocrinol. Metab..

[5] Yeap B.B. (2009). Testosterone and ill-health in aging men. Nat. Clin. Pr. Endocrinol. Metab..

[6] Kelly D.M., Jones T.H. (2015). Testosterone and obesity. Obes. Rev..

[7] Park H.J., Ahn S.T., Moon D.G. (2019). Evolution of Guidelines for Testosterone Replacement Therapy. J. Clin. Med..

[8] Basaria S., Coviello A.D., Travison T.G., Storer T.W., Farwell W.R., Jette A.M., Eder R., Tennstedt S., Ulloor J., Zhang A. (2010). Adverse Events Associated with Testosterone Administration. N. Engl. J. Med..

[9] Vigen R., O’Donnell C.I., Barón A.E., Grunwald G.K., Maddox T.M., Bradley S.M., Barqawi A., Woning G., Wierman M.E., Plomondon M.E. (2013). Association of Testosterone Therapy With Mortality, Myocardial Infarction, and Stroke in Men With Low Testosterone Levels. JAMA.

[10] Martin L.J., Touaibia M. (2020). Improvement of Testicular Steroidogenesis Using Flavonoids and Isoflavonoids for Prevention of Late-Onset Male Hypogonadism. Antioxidants.

[11] Ito A., Shirakawa H., Takumi N., Minegishi Y., Ohashi A., Howlader Z.H., Ohsaki Y., Sato T., Goto T., Komai M. (2011). Menaquinone-4 enhances testosterone production in rats and testis-derived tumor cells. Lipids Heal. Dis..

[12] Ho H.-J., Shirakawa H., Yoshida R., Ito A., Maeda M., Goto T., Komai M. (2016). Geranylgeraniol enhances testosterone production via the cAMP/protein kinase A pathway in testis-derived I-10 tumor cells. Biosci. Biotechnol. Biochem..

[13] Nakayama Y., Ho H.-J., Yamagishi M., Ikemoto H., Komai M., Shirakawa H. (2020). Cysteine Sulfoxides Enhance Steroid Hormone Production via Activation of the Protein Kinase A Pathway in Testis-Derived I-10 Tumor Cells. Molecules.

[14] Horigome S., Maeda M., Ho H., Shirakawa H., Komai M. (2016). Effect of Kaempferia parviflora extract and its polymethoxyflavonoid components on testosterone production in mouse testis-derived tumour cells. J. Funct. Foods.

[15] Banihani S.A. (2018). Ginger and Testosterone. Biomolecules.

[16] Banihani S.A. (2019). Testosterone in Males as Enhanced by Onion (*Allium Cepa* L.). Biomolecules.

[17] Banihani S.A. (2019). Mechanisms of honey on testosterone levels. Heliyon.

[18] Kodera Y., Suzuki A., Imada O., Kasuga S., Sumioka I., Kanezawa A., Taru N., Fujikawa M., Nagae S., Masamoto K. (2002). Physical, Chemical, and Biological Properties of *S*-Allylcysteine, an Amino Acid Derived from Garlic. J. Agric. Food Chem..

[19] Colín-González A.L., Santana R.A., Silva-Islas C.A., Chánez-Cárdenas M.E., Santamaría A., Maldonado P.D. (2012). The Antioxidant Mechanisms Underlying the Aged Garlic Extract- and *S*-Allylcysteine-Induced Protection. Oxidative Med. Cell. Longev..

[20] Colín-González A.L., Ali S.F., Túnez I., Santamaría A. (2015). On the antioxidant, neuroprotective and anti-inflammatory properties of *S*-allyl cysteine: An update. Neurochem. Int..

[21] Agbana Y.L., Ni Y., Zhou M., Zhang Q., Kassegne K., Karou S.D., Kuang Y., Zhu Y. (2020). Garlic-derived bioactive compound *S*-allylcysteine inhibits cancer progression through diverse molecular mechanisms. Nutr. Res..

[22] Asdaq S., Inamdar M. (2010). Potential of garlic and its active constituent, *S*-allyl cysteine, as antihypertensive and cardioprotective in presence of captopril. Phytomedicine.

[23] Chuah S.C., Moore P.K., Zhu Y.Z. (2007). *S*-allylcysteine mediates cardioprotection in an acute myocardial infarction rat model via a hydrogen sulfide-mediated pathway. Am. J. Physiol. Circ. Physiol..

[24] Saravanan G., Ponmurugan P., Gandhipuram P.S., Rajaraja T. (2009). Antidiabetic properties of *S*-allyl cysteine, a garlic component on streptozotocin-induced diabetes in rats. J. Appl. Biomed..

[25] Saravanan G., Ponmurugan P. (2011). Ameliorative potential of *S*-allyl cysteine on oxidative stress in STZ induced diabetic rats. Chem. Interact..

[26] Amano H., Kazamori D., Itoh K., Kodera Y. (2015). Metabolism, Excretion, and Pharmacokinetics of *S*-Allyl-l-Cysteine in Rats and Dogs. Drug Metab. Dispos..

[27] Ried K., Frank O.R., Stocks N.P. (2010). Aged garlic extract lowers blood pressure in patients with treated but uncontrolled hypertension: A randomised controlled trial. Maturitas.

[28] Yang J., Wang T., Rao K., Zhan Y., Chen R.-B., Liu Z., Li M.-C., Zhuan L., Zang G.-H., Guo S.-M. (2013). *S*-allyl cysteine restores erectile function through inhibition of reactive oxygen species generation in diabetic rats. Andrology.

[29] Takemura S., Ichikawa H., Naito Y., Takagi T., Yoshikawa T., Minamiyama Y. (2014). *S*-allyl cysteine ameliorates the quality of sperm and provides protection from age-related sperm dysfunction and oxidative stress in rats. J. Clin. Biochem. Nutr..

[30] Lee A.-S., Lee S.-H., Lee S., Yang B.-K. (2019). Effects of streptozotocin and *S*-allyl-l-cysteine on motility, plasma membrane integrity, and mitochondrial activity of boar spermatozoa. Trop. Anim. Heal. Prod..

[31] Tremblay J.J. (2015). Molecular regulation of steroidogenesis in endocrine Leydig cells. Steroids.

[32] Miller W.L., Bose H.S. (2011). Early steps in steroidogenesis: Intracellular cholesterol trafficking. J. Lipid Res..

[33] Manna P.R., Chandrala S.P., Jo Y., Stocco D.M. (2006). cAMP-independent signaling regulates steroidogenesis in mouse Leydig cells in the absence of StAR phosphorylation. J. Mol. Endocrinol..

[34] Midzak A.S., Chen H., Papadopoulos V., Zirkin B.R. (2009). Leydig cell aging and the mechanisms of reduced testosterone synthesis. Mol. Cell. Endocrinol..

[35] Weng J.-H., Chung B.-C. (2016). Nongenomic actions of neurosteroid pregnenolone and its metabolites. Steroids.

[36] Ratner M.H., Kumaresan V., Farb D.H. (2019). Neurosteroid Actions in Memory and Neurologic/Neuropsychiatric Disorders. Front. Endocrinol..

[37] Imai T., Kosuge Y., Endo-Umeda K., Miyagishi H., Ishige K., Makishima M., Ito Y. (2013). Protective effect of *S*-allyl-l-cysteine against endoplasmic reticulum stress-induced neuronal death is mediated by inhibition of calpain. Amino Acids.

[38] Nishiyama N., Moriguchi T., Morihara N., Saito H. (2001). Ameliorative effect of *S*-allylcysteine, a major thioallyl constituent in aged garlic extract, on learning deficits in senescence-accelerated mice. J. Nutr..

[39] Baluchnejadmojarad T., Kiasalari Z., Afshin-Majd S., Ghasemi Z., Roghani M. (2017). *S*-allyl cysteine ameliorates cognitive deficits in streptozotocin-diabetic rats via suppression of oxidative stress, inflammation, and acetylcholinesterase. Eur. J. Pharmacol..

[40] Ruiz-Sánchez E., Pedraza-Chaverri J., Medina-Campos O.N., Maldonado P.D., Rojas P. (2020). *S*-allyl Cysteine, a Garlic Compound, Produces an Antidepressant-Like Effect and Exhibits Antioxidant Properties in Mice. Brain Sci..

[41] Miller W.L. (2013). Steroid hormone synthesis in mitochondria. Mol. Cell. Endocrinol..

[42] Dyson M.T., Kowalewski M.P., Manna P.R., Stocco D.M. (2009). The differential regulation of steroidogenic acute regulatory protein-mediated steroidogenesis by type I and type II PKA in MA-10 cells. Mol. Cell. Endocrinol..

[43] Park T., Oh J.-H., Jang Y.P., Lee J.H. (2017). Oral Administration of (S)-Allyl-l-Cysteine and Aged Garlic Extract to Rats: Determination of Metabolites and Their Pharmacokinetics. Planta Med..

[44] Khaki A., Fathiazad F., Nouri M., Khaki A.A., Khamenehi H.J., Hamadeh M. (2009). Evaluation of androgenic activity of allium cepa on spermatogenesis in the rat. Folia Morphol..

[45] Lowry O.H., Rosebrough N.J., Farr A.L., Randall R.J. (1951). Protein measurement with the Folin phenol reagent. J. Biol. Chem..

